# Enhanced Magnetic Properties of Co-Doped BiFeO_3_ Thin Films via Structural Progression

**DOI:** 10.3390/nano10091798

**Published:** 2020-09-10

**Authors:** Liang Bai, Mingjie Sun, Wenjing Ma, Jinghai Yang, Junkai Zhang, Yanqing Liu

**Affiliations:** 1 Key Laboratory of Functional Materials Physics and Chemistry of the Ministry of Education, Jilin Normal University, Changchun 130103, China; L20141019@126.com (L.B.); j56751136@163.com (M.S.); mwjsgx1996@163.com (W.M.); jhyang1@jlnu.edu.cn (J.Y.); 2 United Laboratory of High Pressure Physics and Earthquake Science, Institute of Earthquake Forecasting, China Earthquake Administration, Beijing 100036, China

**Keywords:** BiFeO_3_ thin film, crystal structure, magnetic properties

## Abstract

Co^3+^ doping in BiFeO_3_ is expected to be an effective method for improving its magnetic properties. In this work, pristine BiFeO_3_ (BFO) and doped BiFe_1-x_Co_x_O_3_ (BFC_x_O, x = 0.01, 0.03, 0.05, 0.07 and 0.10) composite thin films were successfully synthesized by a sol–gel technique. XRD and Raman spectra indicate that the Co^3+^ ions are substituted for the Fe^3+^ ion sites in the BFO rhombohedral lattice. Raman vibration of oxygen octahedron is obviously weakened due to the lattice distortion induced by the size mismatch between two B-site cations (Fe^3+^ and Co^3+^ ions), which has an impact on the magnetic properties of BFC_x_O. SEM images reveal a denser agglomeration in Co-doped samples. TEM results indicate that the average size of grains is reduced due to the Co^3+^ substitution. XPS measurements illustrate that the replacement of Fe^3+^ with Co^3+^ effectively suppresses the generation of oxygen defects and increases the concentration of Fe^3+^ ions at the B-site of perovskite lattice. Vibrating sample magnetometer (VSM) measurements show that the remanent magnetization (Mr) of BFC_0.07_O (3.6 emu/cm^3^) and the saturation magnetization (Ms) of BFC_0.10_O (48.84 emu/cm^3^) thin film both increase by approximately two times at room temperature, compared with that of the pure BFO counterpart.

## 1. Introduction

**Figure 1:**
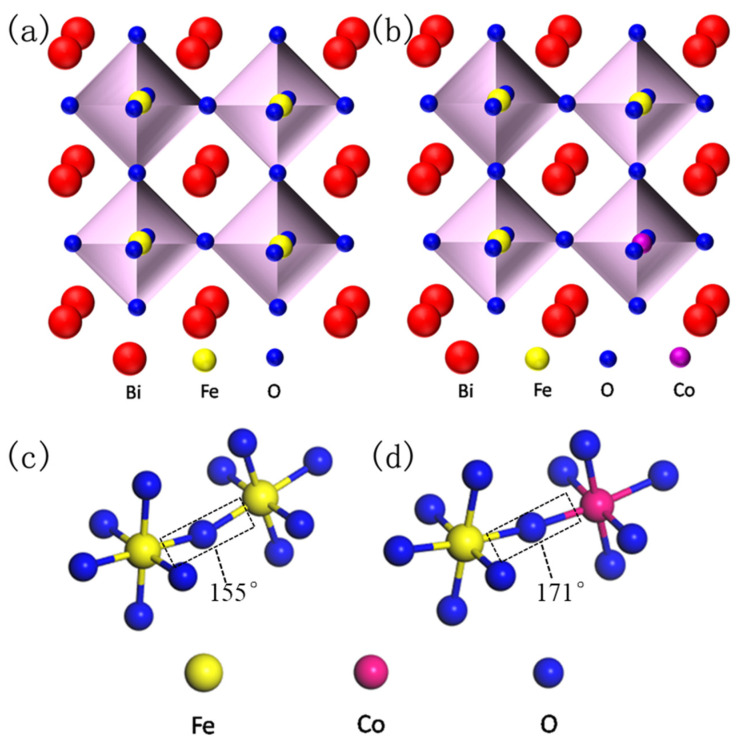
Simulated crystal structure of perovskite BiFeO_3_ (BFO) (**a**) and Co substitution BFO (**b**), and the corresponding super-exchange interaction of Fe–O–Fe (**c**) and Fe–O–Co (**d**).

Recently, magnetoelectric and multiferroic materials have attracted considerable attention due to their abundant physical properties and potential applications in sensors, spintronic devices, memory devices, magnetoelectric devices, capacitors, nonvolatile logic, etc. [1,2]. BiFeO_3_ (BFO), a kind of single crystal multiferroic material, is one of the most well-known compounds of ABO_3_ perovskite, and its G-type structure shows both (anti-) ferromagnetism and ferroelectricity at room temperature [2,3]. BFO always crystallizes with the rhombohedral structure (space group, R3c) where Bi^3+^ ions occupy the A-site and Fe^3+^ ions occupy the B-site in the distorted perovskite lattice [4,5]. It has been reported that the super-exchange interaction between Fe^3p^ and O^2p^ ions plays an important role in the ferromagnetic properties of BFO. The ferroelectric properties stem from the off-center displacement of 6^s2^ lone pair electrons of Bi^3+^ ions [6,7]. Meanwhile, the potential magnetoelectric coupling effect in BFO has also attracted much attention [8,9]. However, BFO always shows a long-range spin cycloid structure (Figure 1) because the impurity phases (such as Bi_2_Fe_4_O_9_, Bi_24_FeO_40_, etc.) are mixed in its rhombohedral phase, thus limiting its potential applications [10,11]. Therefore, great efforts have been made to improve the structures and properties of BFO by means of ions doping, such as A-site doping rare-earth (Er^3+^, La^3+^, Sm^3+^ and Ho^3+^, etc.) or divalent alkaline-earth metal ions (Ba^2+^, Ca^2+^, Sr^2+^, etc.), B-site doping transition metal ions (Cr^3+^, Mn^4+^, Ti^4+^, etc.) and A–B-site co-doping (Ho–Mn, Ce–Zr, Sm–Zr, etc.) [9,10,11,12,13,14,15,16,17].

Trivalent cobalt (Co^3+^) is a typical transition metal ion with favorable magnetic activity. The effective doping of Co^3+^ ions in ferroelectric materials is of great significance for their applications in multiferroic memory devices [17,18]. In this work, BiFe_1-x_Co_x_O_3_ (x = 0.00–0.10) (BFC_x_O) thin films were synthesized on the Si substrates by the sol–gel technique. The structures, surface morphologies, and magnetic properties of BiFe_1-x_Co_x_O_3_ (x = 0.00–0.10) thin films were established by the XRD, XPS, Raman, SEM, TEM and vibrating sample magnetometer (VSM). In particular, the magnetic properties of doped BFC_x_O thin films were significantly improved compared with those of the pristine BFO counterpart. The underlying reasons for the enhanced magnetic properties were also investigated and discussed.

## 2. Experimental Details

**Figure 2:**
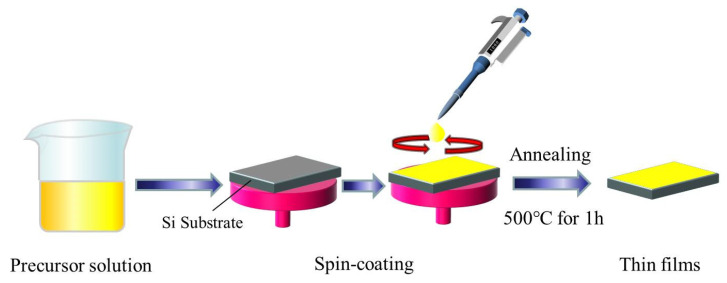
Diagram of the preparation process for pure BiFeO_3_ (BFO) and BiFe_1-x_Co_x_O_3_ (BFC_x_O, x = 0.01, 0.03, 0.05, 0.07 and 0.10) composite thin films.

Pure BiFeO_3_ (BFO) and BiFe_1-x_Co_x_O_3_ (BFC_x_O, x = 0.01, 0.03, 0.05, 0.07 and 0.10) composite thin films were fabricated on Si substrates via the sol–gel technique. High-purity bismuth nitrate [Bi(NO_3_)_3_·5H_2_O] (Shanghai, China), ferric nitrate [Fe(NO_3_)_3_·9H_2_O] (Shanghai, China) and Cobalt(II) nitrate hexahydrate [Co(NO_3_)_2_·6H_2_O] (Shanghai, China) were used as the starting materials. These agents were mixed with ethylene glycol in a specific ratio and stirred thoroughly until clarified. After aging for 24 h, a homogeneous precursor solution of 0.2 mol/L was obtained. The uniform precursor solution grew on an impurity-free silicon substrate via the spin-casting method. The spin-coating process was divided into two steps of 500 rpm for 3 s and 4000 rpm for 20 s, and subsequently, the wet homogeneous films were pre-annealed at 350 °C for 6 min. The above-mentioned process was repeated by spin-coating each layer to obtain a semi-finished film of moderate thickness. Finally, all semi-finished samples were crystallized in air at 500 °C for 1 h and then cooled to room temperature to obtain perfect thin films. The diagram of the experimental procedure is shown in Figure 2.

The structural characteristics and phase purity of all thin films were analyzed by X-ray diffraction patterns (XRD) (XRD, Rigaku Corporation, Tokyo, Japan) at room temperature using Cu Kαradiation (40 kV, 200 mA). The surface morphologies of these samples and the thickness of cross-sections were characterized by the field emission scanning electron microscopy (FESEM) (Hitachi S-4800, JEOL Ltd., Tokyo, Japan) Model Hitachi, S-570. The interplanar spacing of the samples was investigated by transmission electron microscope (TEM, 200 keV, JEM-2100HR, JEOL Ltd., Tokyo, Japan). A Lake Shore 7407 vibrating sample magnetometer (VSM) (VSM, Lake Shore, Columbus, OH, USA) was used to investigate the magnetic hysteresis (M–H) loops of the samples at room temperature. Raman spectra were measured by a Renishaw MicroRaman spectrometer (Renishaw, London, UK) equipped with an Ar ion laser excitation at 514 nm. X-ray photoelectron spectra (XPS) were conducted via A1 Ka line by using a Thermo Scientific ESCALAB 250Xi A1440 system (XPS, Thermo Fisher Scientific, Waltham, MA, USA).

## 3. Results and Discussion

In order to facilitate the understanding about the doping principle and super-exchange interaction of BFO, the crystal structures of pure BFO and doped BFC_x_O are simulated as seen in Figure 1a–d. As is commonly known, the BFO composite thin film possesses a distorted rhombohedral perovskite structure (space group R3c). FeO_6_ octahedron is composed of iron ions at the center and six oxygen ions in the surrounding areas. Additionally, bismuth ions (the apex position) form a cube in which FeO_6_ octahedron is embedded. Fe^3+^ ions occupy the center of the body and oxygen ions occupy the center of the surface. In Co^3+^-doped BFO thin films, two adjacent FeO_6_/CoO_6_ octahedrons are linked by sharing one oxygen anion [19]. Doping Co^3+^ ions will lead to the random replacement of some Fe^3+^ ions at B-site, thus inhibiting the generation of the spin cycloid spiral modulation structure due to the size effect and chemical strain effect caused by the size mismatch of the two B-site cations (Fe^3+^ and Co^3+^ ions) [20]. Figure 1c,d clearly illustrates that Co ions successfully replace Fe ions and form high-energy Co–O bonds, leading to an increase in the inclination angle of the super-exchange interaction [21]. The super-exchange interaction of Fe–O–Fe (or Co) is improved, which is beneficial to enhancing the magnetic properties of BFC_x_O (see details below).

**Figure 3:**
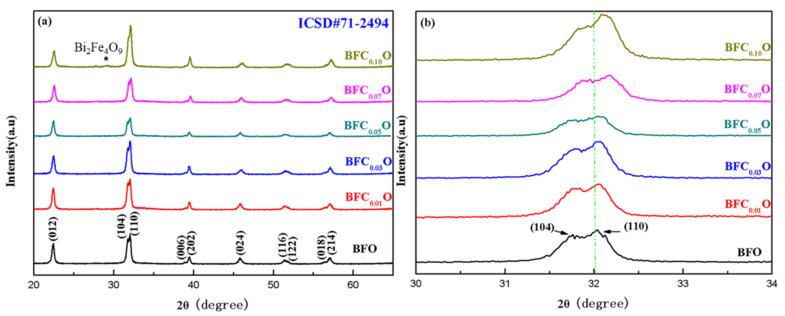
(**a**) XRD patterns of BFO and BFC_x_O thin films, and (**b**) correspondingly magnified patterns around 32°.

Figure 3a,b shows the X-ray diffraction (XRD) patterns of the BiFe_1-x_Co_x_O_3_ (BFC_x_O, x = 0.00, 0.01, 0.03, 0.05, 0.07 and 0.10) thin films. The diffraction peaks of all the samples are completely consistent with the standard XRD pattern of the rhombohedral phase (space group R3c) (JCPDS # 71-2494). Meanwhile, a weak XRD peak of impurity is found at 28.8° corresponding to Bi_2_Fe_4_O_9_ [17,21]. Bi_2_Fe_4_O_9_ will have no significant impact on the intrinsic structures and properties of BFC_x_O because it is paramagnetic and its content is relatively small in the samples [17,21]. Figure 3b shows magnified XRD patterns of Co substituted thin films at different concentrations in the vicinity of 2θ = 32°. Obviously, the diffraction peaks of (104) and (110) move at higher angles with the increase of Co^3+^ concentration, indicating that the Co ions have been successfully positioned in the host lattice of BFO. The radius of Co^3+^ ions is smaller than that of Fe^3+^ ions, which will lead to lattice contraction and structural distortion.

**Figure 4:**
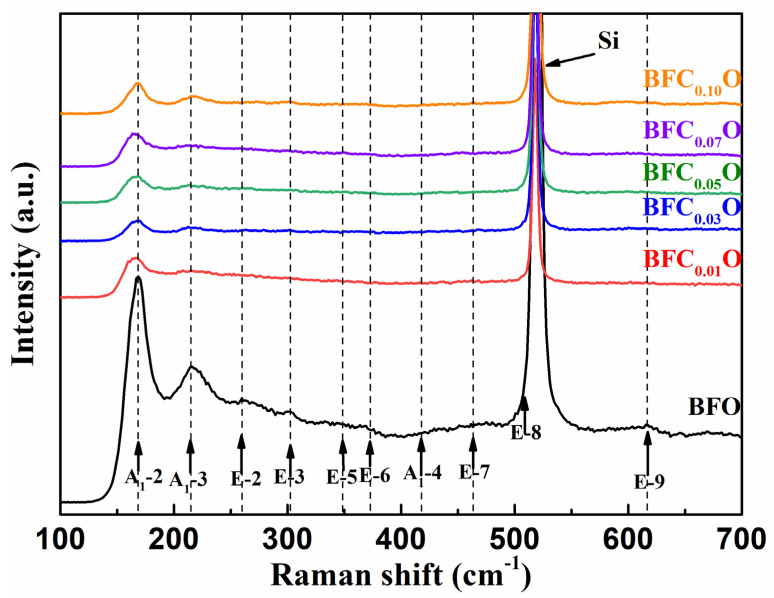
Raman spectra of BFO and BFC_x_O thin films at room temperature.

To further investigate the structural changes of all thin films with doping concentrations, we carried out Raman spectra measurements on BFO and BFCxO thin films, as shown in Figure 4. According to group theory, BFO displays 13 optical-phonon active modes (4A_1_ + 9E) with space group R3c [22,23]. The 4 modes at 149.6, 170.0, 217.6 and 436.1 cm^−1^ can be designated as A_1_–symmetry longitudinal optical modes [A_1_-1, A_1_-2, A_1_-3, A_1_-4 (LO)], and 8 modes at 77.0, 265.6, 318.7, 345.4, 369.2, 482.3, 515.6 and 617.8 cm^−1^ are associated with transverse optical E(TO) (E-1, E-2, E-3, E-5, E-6, E-7, E-8, E-9 (TO)) phonons [24,25]. In this work, the E-8 mode of BFO thin film overlaps with the Raman vibration mode of silicon substrate. The results of first-principles calculation have revealed that the Bi-O vibrations only participate in the low-frequency region of the A_1_ modes and the Fe–O vibrations mainly participate in the high-frequency region of the E modes [24,25,26,27]. Compared with that of pure BFO, the peak positions of Raman spectra of BFCxO are slightly red-shifted, and the intensity decreases as Co doping concentration increases. Among them, the weakening of A_1_-2 and A_1_-3 modes is due to the relatively weak Bi 6s^2^ lone pair electrons in the doped BFC_x_O thin films in stereochemistry, which also indicates that the Co ions are successfully located into the BFO lattice. The high-frequency modes are mainly attributed to the vibration of FeO_6_ octahedron. When the Fe sites are occupied by Co^3+^ ions, Co^3+^ ions cannot completely fill the space of Fe^3+^ ions due to the size mismatch. Therefore, the oxygen octahedrons are greatly distorted by the Jahn–Teller effect [28,29]. This is caused by the change in Fe–O bonds and accounts for the enhanced magnetic properties to be described as follows.

**Figure 5:**
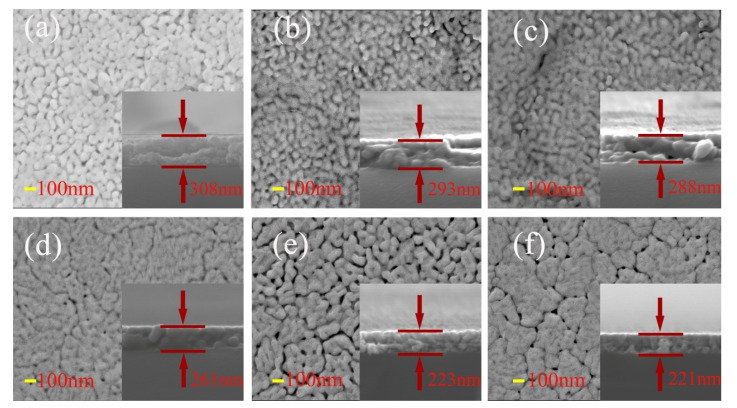
SEM images of surface and cross-sectional morphologies of (**a**) BFO, (**b**) BFC_0.01_O, (**c**) BFC_0.03_O, (**d**) BFC_0.05_O, (**e**) BFC_0.07_O and (**f**) BFC_0.10_O thin films.

**Figure 6:**
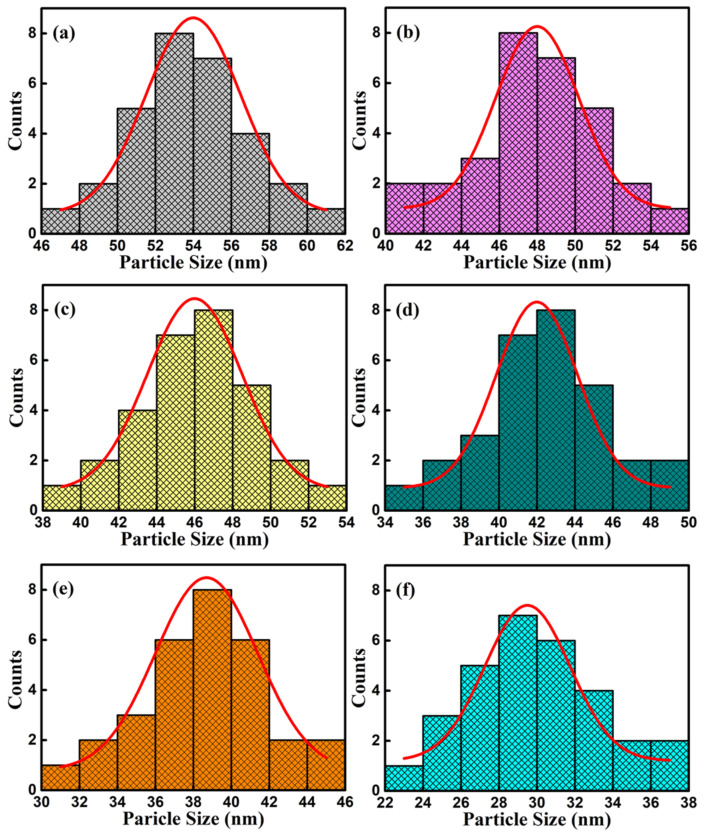
Histograms for particle size distribution and Gaussian fitting curve of (**a**) BFO, (**b**) BFC_0.01_O, (**c**) BFC_0.03_O, (**d**) BFC_0.05_O, (**e**) BFC_0.07_O and (**f**) BFC_0.10_O thin films.

The SEM images of surface and cross-section of BFC_x_O thin films are shown in Figure 5a–f. It can be seen that the Co-substituted thin films not only show a homogeneous surface morphology and conspicuous agglomeration, but also attach well to the silicon substrates without obvious separation. Additionally, the cross-sectional thickness of BFC_x_O thin films is 308, 293, 288, 261, 223 and 221 nm, respectively. With the increase of Co doping concentration, the thickness obviously decreases, indicating that Co doping makes the surface uniformly dense and grain size decreases [26,27]. The grain size histogram and Gaussian fitting also prove that the grain size decreases with the increase of the Co concentration, as shown in Figure 6. It is also caused by the smaller radius of the Co ions, and correspondingly, the lattice collapses and oxygen vacancies are formed [26,27,28,29]. The suppression of oxygen vacancy concentration will slow down the movement of oxygen ions and the growth rate of crystal grains, thus reducing the size of crystal grains [26].

**Figure 7:**
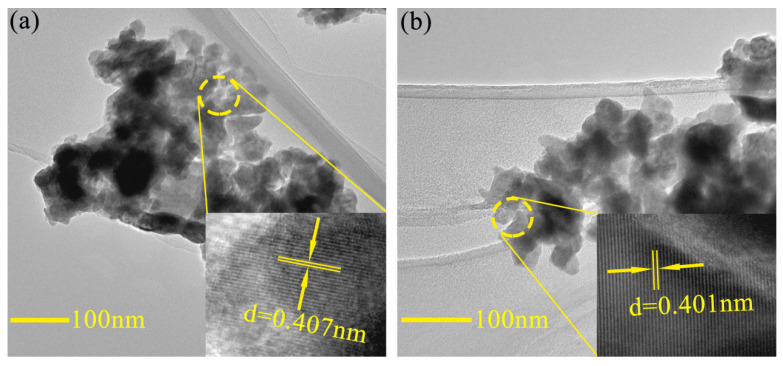
Typical TEM images of (**a**) BFO and (**b**) BFC_0.05_O thin film. The insets are high resolution TEM images of BFO and BFC_0.05_O thin film.

Figure 7 shows the typical TEM and HRTEM images of BFO and BFC_0.05_O thin films. The grain size is reduced slightly with the doping of Co^3+^ ions, which is consistent with the results of SEM images. The HRTEM images are shown in the inset of Figure 7, which are derived from the region within the circle. The lattice spacing of the typical crystalline region is about 0.407 and 0.401 nm, respectively, which is in good agreement with the (012) lattice plane of pure BFO (#71-2494).

**Figure 8:**
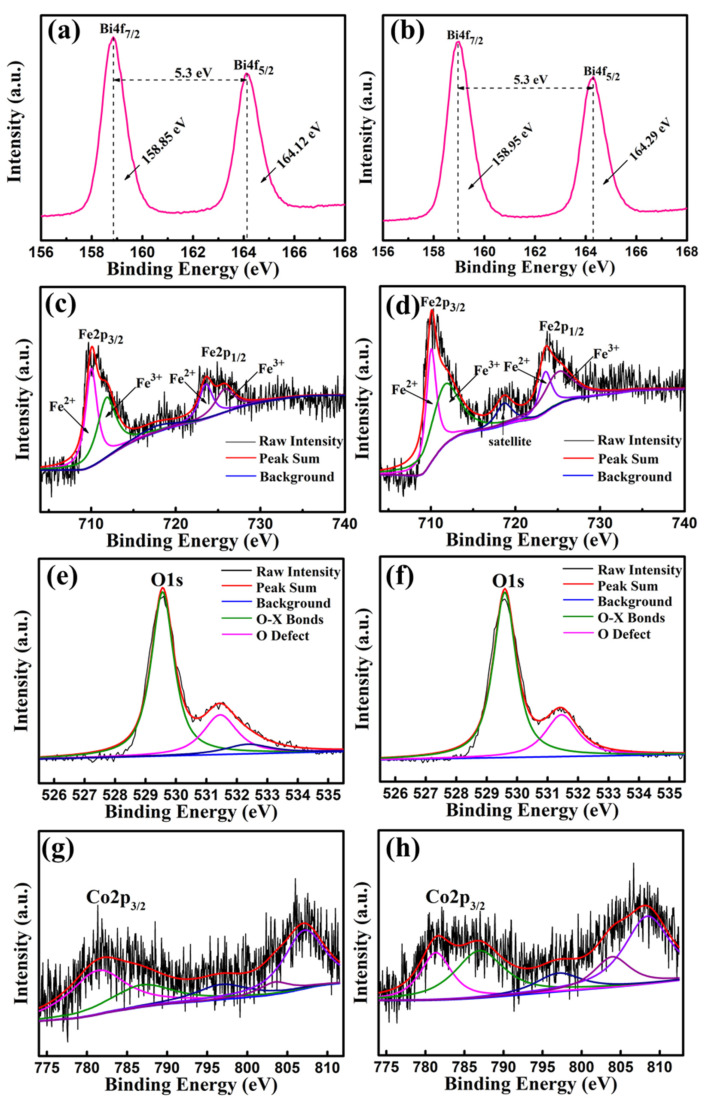
XPS spectra of the as-annealed BFC_0.01_O thin films in the binding energy regions of (**a**) Bi4f, (**c**) Fe2p, (**e**) O1s and (**g**) Co2p; XPS spectra of the as-annealed BFC_0.07_O thin films in the binding energy regions of (**b**) Bi4f, (**d**) Fe2p, (**f**) O1s and (**h**) Co2p.

The Bi4f, Fe2p, O1s and Co2p peaks of BFC_0.01_O and BFC_0.07_O thin films were measured and analyzed by XPS, as shown in Figure 8a–h. The difference in energy between Bi4f_7/2_ and Bi4f_5/2_ in Figure 8a,b is 5.3 eV, which is a standard value of Bi–O bonding. Figure 8c,d shows the Fe2p peaks of BFC_0.01_O and BFC_0.07_O thin films, respectively. The Fe2p_3/2_ peak position of BFC_0.01_O and BFC_0.07_O thin films is 710.06 and 710.11 eV, respectively, both of which are within the reasonable range (710.06–711.2 eV) [26,27]. The peak positions of Fe2p_1/2_ are 723.6 and 723.7 eV, respectively. The difference in binding energy (Spin-orbit splitting energy) between Fe2p_3/2_ and Fe2p_1/2_ peaks is 13.0 and 13.6 eV, respectively, agreeing well with the standard value of 13.6 eV [30]. The Fe2p_3/2_ and Fe2p_1/2_ peaks are fitted by two portions of Fe^3+^ ions and Fe^2+^ ions, and the ratios of Fe^3+^/Fe^2+^ of the BFC_0.01_O and BFC_0.07_O film samples are determined to be 0.57 and 1.59, respectively, indicating that there are more Fe^3+^ ions in the samples [28,29,30]. A satellite peak is found at the binding energy of 718.6 eV in Figure 8d, which is mainly related to the oxidation state of Fe and is determined by the difference in binding energy between the satellite peak and the 2p_3/2_ principal peak (Fe^2+^ ~ 6 eV, Fe^3+^ ~ 8 eV) [30,31,32,33]. The difference in binding energy between them is greater than 8 eV, indicating that the valence state of Fe is mainly +3 valences in the BFC_0.07_O sample [30,31,32,33]. Therefore, Co doping is expected to suppress the formation of Fe^2+^ ions in BFC_x_O, thereby improving the magnetic properties [29,30,31,32]. Due to the application of the sol–gel technique, Figure 8e,f shows the unavoidable O1s XPS spectra of oxygen vacancies. The O1s XPS peak is divided into two distinct peaks, which correspond to O^2−^ ions in the X–O bonds and O defects in the structure, respectively [32,33]. The oxygen vacancy concentrations (O defects/O^2−^ ions ratio) of BFC_0.01_O and BFC_0.07_O thin films significantly decrease from 0.48 to 0.37, suggesting that Co doping can restrain the formation of oxygen defects, which is consistent with previously reported results [32,33]. As seen in Figure 8g,h, the binding energy values of the Co2p_3/2_ XPS spectra are observed at 781.94 and 781.6 eV, corresponding to the standard peak positions of Co^3+^ ions [34].

**Figure 9:**
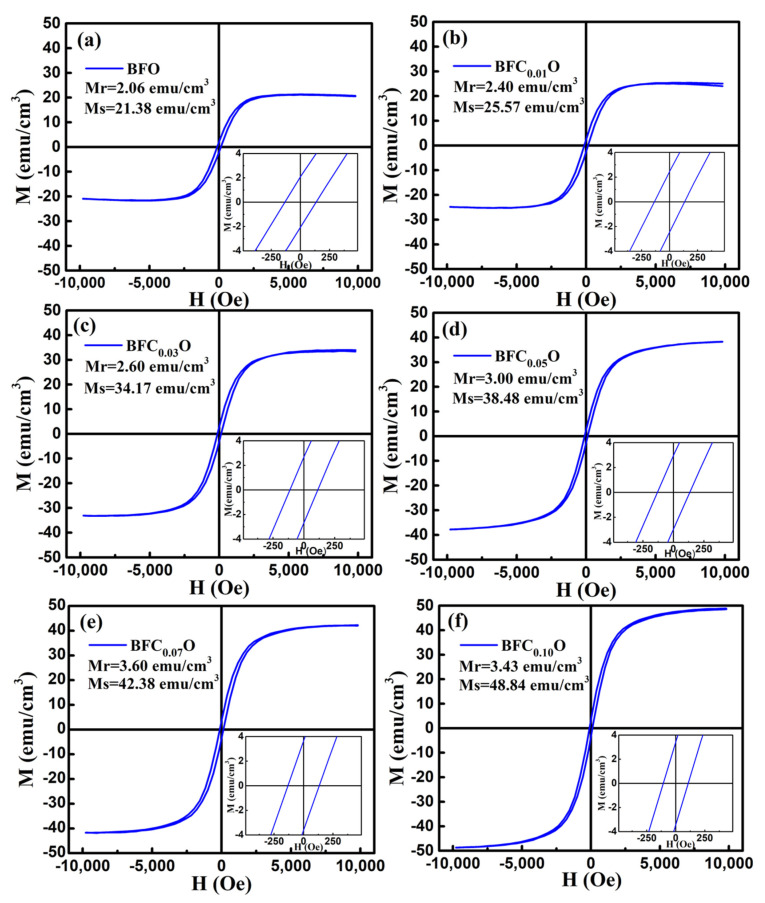
The magnetic hysteresis (M–H) loops for (**a**) BFO, (**b**) BFC_0.01_O, (**c**) BFC_0.03_O, (**d**) BFC_0.05_O, (**e**) BFC_0.07_O and (**f**) BFC_0.10_O thin film at room temperature.

**Figure 10:**
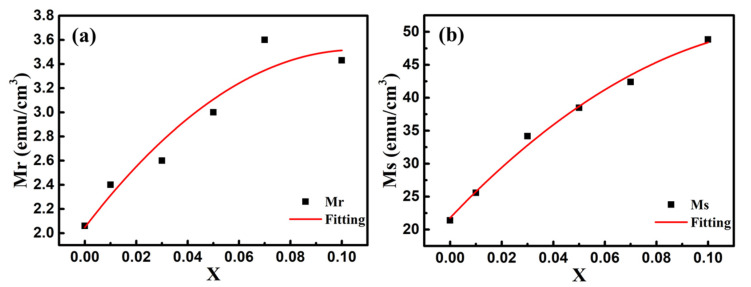
The changes of (**a**) remanent magnetization (Mr) and (**b**) saturation magnetization (Ms) values with Co doping concentration. Red lines are the simulated results according to the binomial polynomial equation.

In order to explore the impact of Co doping on the magnetic properties at room temperature, we carried out the VSM measurements on BFO and BFC_x_O thin films in the magnetic field from 0 to 10 k Oe. The saturated magnetic hysteresis (M–H) loops of BFO and Co-doped thin films are shown in Figure 9a–f. With the increase of the Co doping concentration, the values of both saturation magnetization (Ms) and remanent magnetization (Mr) significantly increased. The values of Ms and Mr for BFO, BFC_0.01_O, BFC_0.03_O, BFC_0.05_O, BFC_0.07_O and BFC_0.10_O are 21.38 and 2.06, 25.57 and 2.40, 34.17 and 2.60, 34.48 and 3.00, 42.38 and 3.60, and 48.84 and 3.43 emu/cm^3^, respectively. The changes of Ms and Mr with the Co doping concentration are shown in Figure 10. Ms always increases linearly but Mr increases first and then decreases with the increase of Co^3+^ concentration over x = 0.10. The maximum value of Mr is 3.60 emu/cm^3^ when the concentration of doped Co is 0.07. Additionally, the maximum value of Ms is 48.84 emu/cm^3^ as the doping concentration is up to 0.10. According to the Goldschmidt tolerance factor t [26,27,28,29],

(1) $$t=\;\frac{R_{A}+R_{O}}{\sqrt{R_{B}+R_{O}}}$$

With the increase of the Co doping concentration, the tolerance factor becomes smaller and less than the unity. This will cause the shrinkage strain of the Fe–O bond, thus increasing the bond angle of FeO_6_ octahedron and increasing the degree of distortion between two adjacent octahedrons [26,27,28,29,33]. In the first principle, the inclination of the Fe^3+^ ions spins can be derived from the formula of the Dzyaloshinskii–Moriya (DM) [33,34] interaction energy of Fe^3+^ ions (H_DM_), which is defined as follows:

(2) $$H_{DM}=\sum_{n=0}^{n}\vec{D_{n}}\cdot \left(\vec{S_{0}}\times \vec{S_{n}}\right)$$

(3) $$\vec{D_{n}}=V_{0}\left[\vec{r_{n-0}}\times \vec{r_{n-n}}\right]$$

where $\vec{D_{n}}$ is the interaction parameter, $V_{O}$ is the microscopic constant [33,34] and $\vec{r_{n-0}}$, $\vec{\;r_{n-n}}$ are the position vectors of the nearest neighbor magnetic ions from the nth O ions to the nearest magnetic Fe ions. $\vec{S_{0}}$ and $\vec{S_{n}}$ are the vectors of the magnetic moments of two Fe ions [34,35]. In an ideal perovskite structure, the bond angle (φ) of Fe–O–Fe is 180°, so the value of $\vec{r_{n-0}}\times \vec{r_{n-n}}$ is zero, as is the value of $H_{DM}$. When  φ begins to deflect from the ideality of 180°, the anti-symmetric exchange energy term $H_{DM}$ will increase. In summary, several possible factors may account for the enhancement of magnetic properties for Co-doped BFC_x_O thin films: (i) an increased Fe–O–Fe spin angle results in the net macroscopic magnetization [25,36,37,38]; (ii) the suppression of the spin cycloid spiral modulation structures will release magnetism [25,26,27,28,29,39]; (iii) enhanced ferromagnetic properties are attributed to the super-exchange interaction between both d^6^ and d^5^ electronic configurations of Co^3+^ and Fe^3+^ [25,26,27,28,29,39]; (iv) Fe^3+^ ions are randomly replaced by Co^3+^ ions, resulting in the non-compensation of the spins on the surface of grains and making grain size closer to or less than the cycloidal modulation wavelength of ~62 nm [25,26,27,28,29,39,40,41,42]. Accordingly, it is an important way to improve the magnetic behavior of BFO thin films, which is beneficial for the application in subminiature devices and information storage.

## 4. Conclusions

In this work, a series of Co-doped BFO thin films were successfully prepared via a facile sol–gel technique. The relationships of structures, surficial morphologies, as well as magnetic properties of the samples were systematically studied. XRD and Raman spectra confirm that all samples are the rhombohedral perovskite structure and Co^3+^ ions are successfully positioned to the host lattice of BFO. SEM and TEM images suggest that a Co doping agent is beneficial for the uniformity of particle formation and the decrease of grain size. The surfaces of Co substitution thin films are attached to a few conspicuous agglomerations with a smaller grain size, which are beneficial for the magnetism release. XPS analysis illustrates that the replacement of Fe^3+^ ions with Co^3+^ ions can suppress the generation of oxygen defects and increase the concentration of Fe^3+^ ions occupying the B-site of perovskite lattice. VSM measurements indicate that the magnetic properties can be improved significantly and the maximum values of Mr and Ms are 3.60 and 48.84 emu/cm^3^, respectively, belonging to BFC_0.07_O and BFC_0.10_O thin films. The obvious enhancements of Ms and Mr mainly derive from the decrease of oxygen defects concentration, the denser agglomerations, the enhanced super-exchange interaction and the breakdown of spiral spin structure. These results demonstrate that Co^3+^ doping in BFO thin films is an effective method to improve their magnetic characteristics. Co-doped BFO thin films should have better application prospects in multiferroic memory devices, information storage and magnetic switch devices at room temperature.
